# Importance of Casual Leisure Activities on Cognitive Health in Older Adults: The Health and Retirement Study

**DOI:** 10.1093/geroni/igaf122.2742

**Published:** 2025-12-31

**Authors:** Jungsu Ryu

**Affiliations:** Marshall University, Huntington, West Virginia, United States

## Abstract

The cognitive health of older adults has become an increasing concern in our society, prompting ongoing research into factors that may positively influence it. This study aimed to examine how different types of casual leisure activities predict cognitive health in older adults. Participants (N = 5152, 59.1% female) came from a subsample of the Health and Retirement Study (HRS) and completed the psychosocial leave-behind questionnaire in 2018. Ages ranged from 50 to 102 years (M = 68.09, SD = 10.27). Descriptive statistics indicated that older adults typically engage in watching TV almost daily, reading several times a week, and walking approximately once a week. A two-step hierarchical regression analysis revealed that seven out of ten leisure activities significantly predicted cognitive health. Specifically, charity work, reading, doing word games, gardening/maintenance, baking or cooking, and walking for 20 minutes were positively associated with cognitive health. In contrast, watching TV was negatively related to cognitive health. Among these activities, reading emerged as the strongest predictor of cognitive well-being in older adults. While previous studies have shown mixed findings regarding the impact of watching TV on cognitive health, this study emphasizes the negative relationship between watching TV and cognitive health. Although confounding factors, such as demographic variables, may influence these findings, the results highlight the importance of engaging older adults in more stimulating and active leisure activities, such as reading and walking, to maintain cognitive health. Reducing passive activities, particularly watching TV, may be beneficial if it replaces more cognitively engaging pursuits.

